# Supplementary material for: Nucleoside-modified VEGFC mRNA induces organ-specific lymphatic growth and reverses experimental lymphedema
Source: Nat Commun. 2021 Jun 8;12:3460. doi: 10.1038/s41467-021-23546-6 (PMC8187400; doi:10.1038/s41467-021-23546-6)
Supplement: Supplementary file 1 — Supplementary Information [file 41467_2021_23546_MOESM1_ESM.pdf]

Nucleoside-Modified VEGFC mRNA Induces Organ-Specific Lymphatic Growth and Reverses Experimental Lymphedema

Dániel Szőke[1,2], Gábor Kovács[1,2], Éva Kemecsei[1,2], László Bálint[1,2], Kitti Szoták-Ajtay[1,2], Petra Aradi[1,2], Andrea Styevkóné Dinnyés[1,2], Barbara L. Mui[3], Ying K. Tam[3], Thomas D. Madden[3], Katalin Karikó[4], Raghu P. Kataru[5], Michael J. Hope[3], Drew Weissman[6], Babak J. Mehrara[5], Norbert Pardi[6,*] and Zoltán Jakus[1,2,*]

[1] Department of Physiology, Semmelweis University School of Medicine, Budapest, Hungary
[2] MTA-SE „Lendület” Lymphatic Physiology Research Group of the Hungarian Academy of Sciences and the Semmelweis University, Budapest, Hungary
[3] Acuitas Therapeutics, Vancouver, BC, Canada
[4] BioNTech RNA Pharmaceuticals, Mainz, Germany
[5] Department of Surgery, Division of Plastic and Reconstructive Surgery, Memorial Sloan Kettering Cancer Center, New York, NY, USA
[6] University of Pennsylvania, Perelman School of Medicine, Philadelphia, PA, USA

* Corresponding authors

Supplementary Figure 1
Szőke et al.

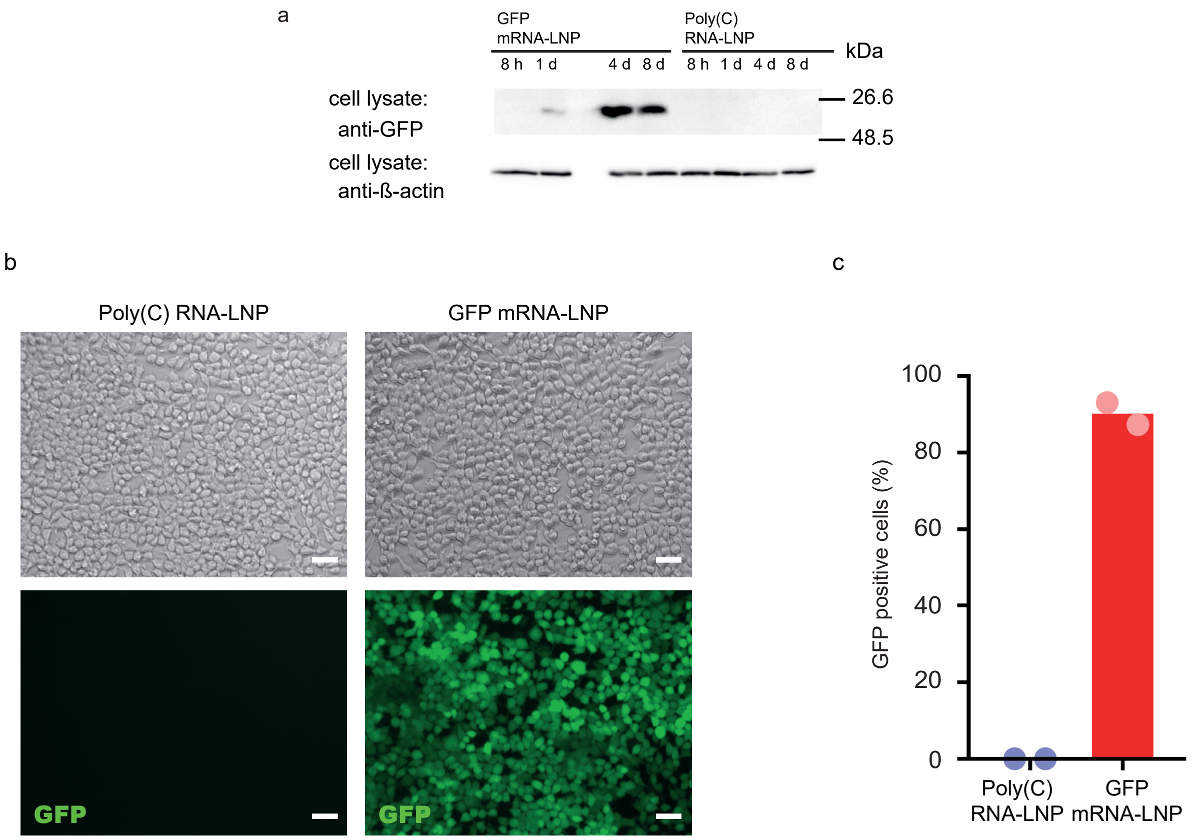

Supplementary Figure 1. GFP protein expression induced by GFP mRNA-LNP treatment in vitro.
(a) 1 µg of Poly(C) or GFP mRNA-LNPs were added to the medium of HEK293T cells, and GFP and β-actin protein expression levels were determined by Western blot analyses. Representative images at different time points are shown of 2 independent experiments. **(b)** GFP expression in HEK293T cells treated with 1 µg of GFP mRNA-LNPs as detected by fluorescent microscopy. Representative images are shown (bars, 50 µm). Two independent experiments were performed. **(c)** Quantitative data for the number of GFP positive HEK293T cells after treatment with Poly(C) or GFP mRNA-LNPs. Data are represented as mean of 2 independent experiments in each group.

Supplementary Figure 2
Szőke et al.

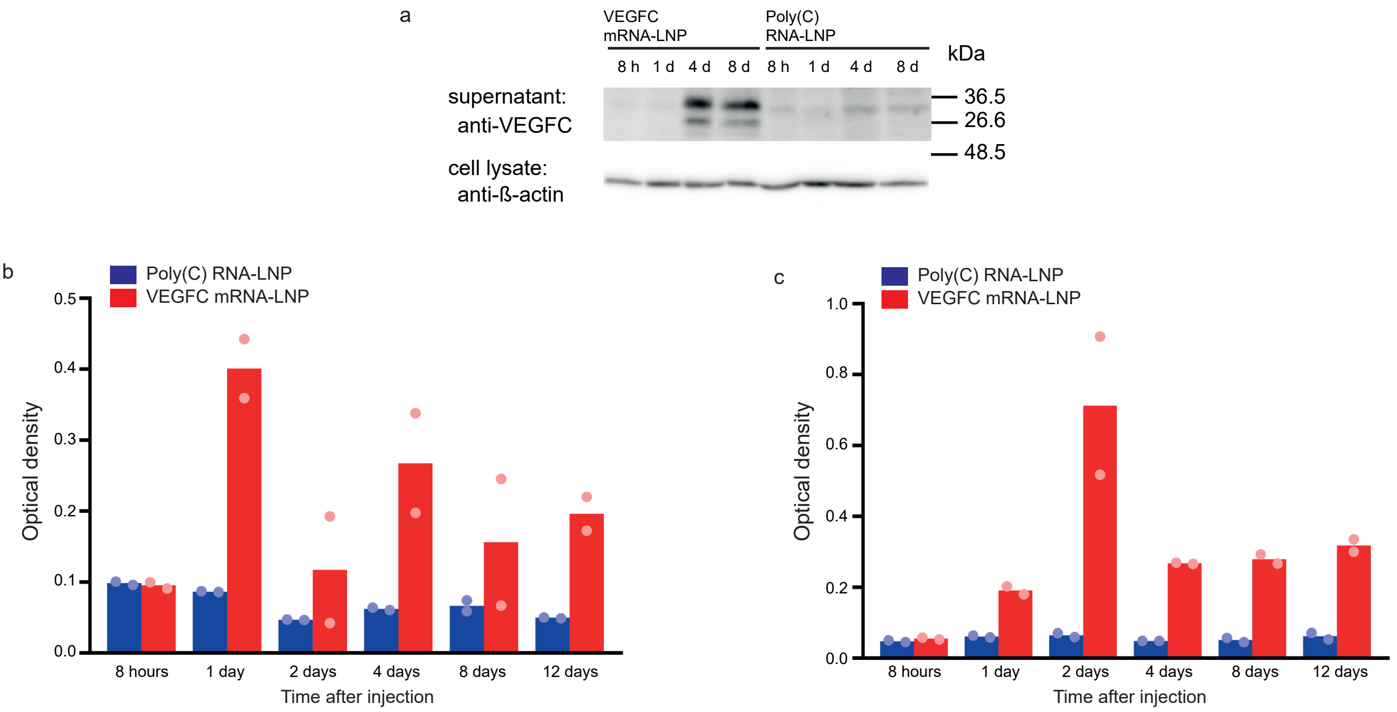

Supplementary Figure 2. Administration of VEGFC mRNA-LNPs induces VEGFC secretion in vitro.
(a) 1 µg of Poly(C) or VEGFC mRNA-LNPs were added to the medium of HEK293T cells, and VEGFC (in supernatants) and β-actin (in cell lysates) protein expression levels were determined at 8 hours and at days 1, 2, 4, 8 and 12 by Western blot analyses. Representative images are shown of 3 independent experiments. **(b-c)** 1 µg of Poly(C) or VEGFC mRNA-LNPs were added to the medium of HEK293T cells, and VEGFC protein expression levels of cell lysates (b) and supernatants (c) were determined by ELISA at 8 hours and at days 1, 2, 4, 8 and 12. Quantitative data for VEGFC amount are represented as mean of 2 experiments.

Supplementary Figure 3
Szőke et al.

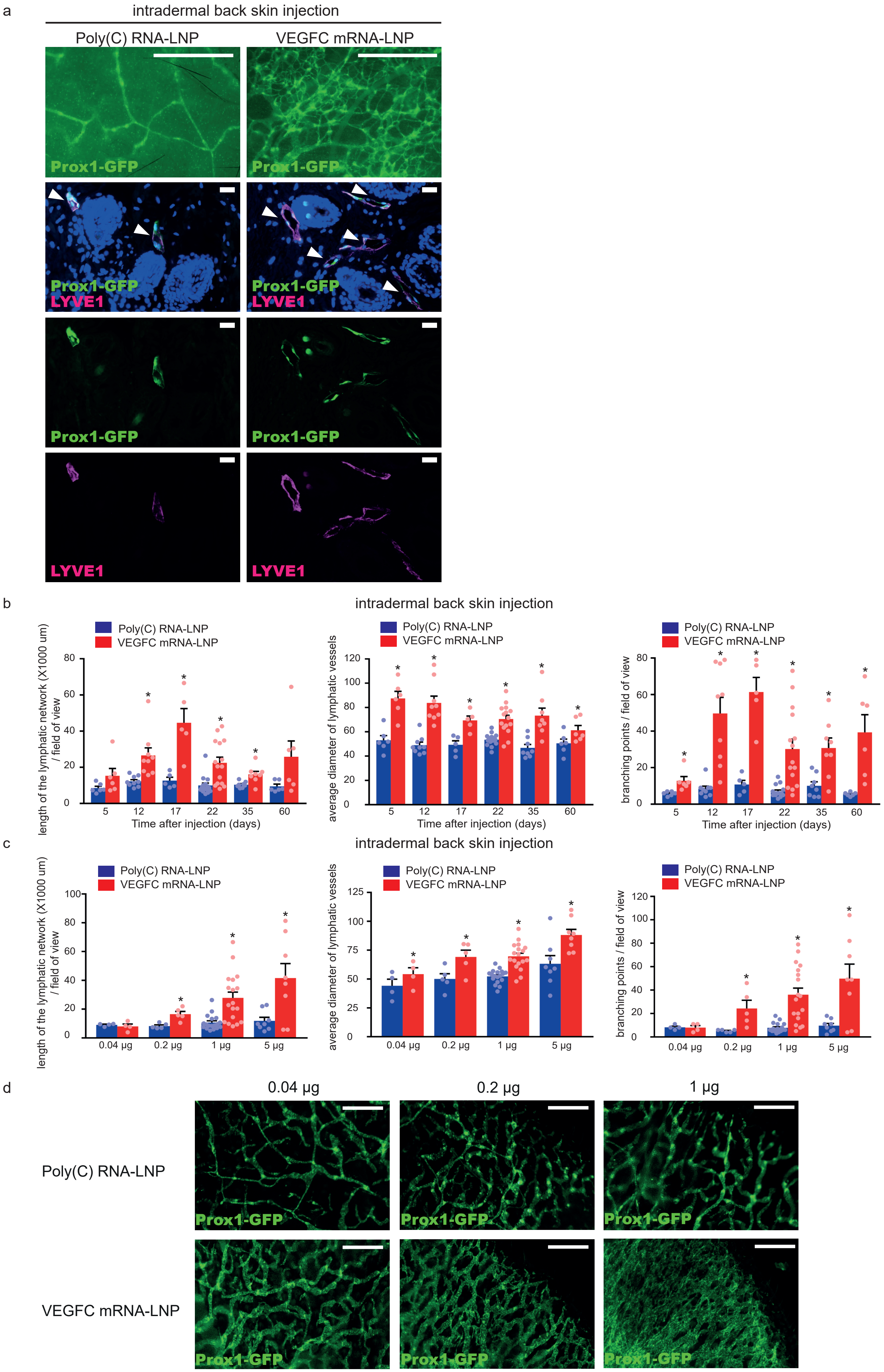

Supplementary Figure 3. Administration of VEGFC mRNA-LNPs induces local lymphatic growth in vivo in back skin and ear.

(a) Analysis of lymphatic morphology in the back skin of lymphatic reporter animals injected with 1 µg of Poly(C) or VEGFC mRNA-LNPs. Representative images of 15 mice 22 days after the treatment are shown by whole-mount fluorescent stereo microscopy (upper panels; bars, 1000 µm) and anti-LYVE1 immunostaining of slides processed by paraffin-based histology (lower panels; bars, 50 µm). Arrows indicate LYVE1 and Prox1-GFP positive lymphatic vessels. (**b)** Assessment of the time-dependent effect of intradermal administration of 1 µg of Poly(C) or VEGFC mRNA-LNPs at days 5, 12, 17, 22, 35 and 60 in the back skin. Quantitative data for the length of lymphatic network, average lymphatic vessel diameter and number of branching points are represented as mean and SEM from LNP-injected back skin of 5-15 mice in each group. (two-tailed, paired T-test, for lymphatic network length P= 0.0009 after 22 days of 15 mice and P= 0.0884 after 60 days of 6 mice. For average lymphatic vessel diameter P= 0.0002 after 22 days of 15 mice and P= 0.0103 after 60 days of 6 mice. For number of branching points P= 0.0010 after 22 days of 15 mice and P= 0.0520 after 60 days of 6 mice.) **(c)** Monitoring the dose-dependent effect of Poly(C) and VEGFC mRNA-LNPs (0.04, 0.2, 1 and 5 µg) 22 days after intradermal treatment of the back skin. Quantitative data for the length of lymphatic network, average lymphatic vessel diameter and number of branching points are represented as mean and SEM from mRNA-LNP complex-injected back skin of 4-18 mice in each group. (two-tailed, paired T-test, for lymphatic network length P= 0.0002 of 18 mice, average lymphatic vessel diameter P= 2.32×10^{-5} of 18 mice and for number of branching points P= 9.64×10^{-5} of 18 mice when injected with 1 µg of Poly(C) or VEGFC mRNA-LNP). **(d)** Monitoring the dose-dependent effect of Poly(C) and VEGFC mRNA-LNPs (0.04, 0.2 and 1 µg) 22 days after intradermal treatment of the ear. Representative images of Poly(C) and VEGFC mRNA-LNP injected ears of 6-17 *Prox1^{GFP}* mice in each group are shown by stereomicroscopy (bars, 500 µm). Asterisks indicate P< 0.05 compared with control. All cell nuclei are labelled with DAPI (blue) in paraffin-embedded tissues.

Supplementary Figure 4
Szőke et al.

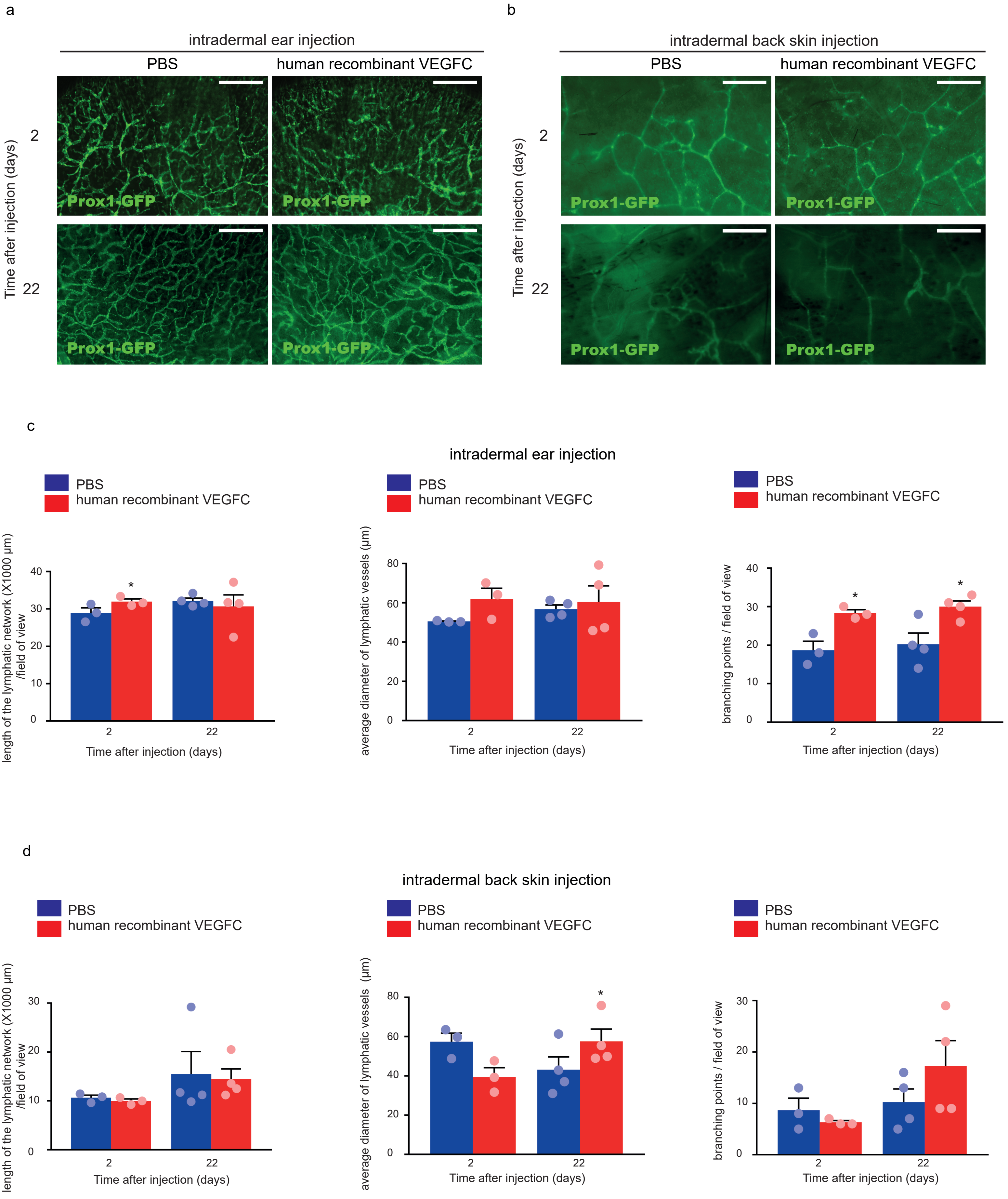

Supplementary Figure 4. VEGFC protein injection induces modest lymphatic growth in mice.

(a-b) The effect of PBS and 1 µg of human recombinant VEGFC intradermal injection on the growth of lymphatic vessels in the ear (a) and back skin (b) 2 and 22 days after the treatment of *Prox1*^{GFP} animals. Representative images by fluorescent stereo microscopy are shown of 4 mice per group (bars, 1000 µm). **(c-d)** Quantitative data for the length of lymphatic network, average lymphatic vessel diameter and number of branching points in PBS or 1 µg of human recombinant VEGFC-treated ears and back skins of *Prox1*^{GFP} animals 2 days (two-tailed, paired T-test, for lymphatic network length P= 0.0479 in ears of 3 mice and P= 0.2371 in back skins of 3 mice, for average lymphatic vessel diameter P= 0.1626 in ears of 3 mice and P= 0.1847 in back skins of 3 mice, for number of branching points P= 0.0317 in ears of 3 mice and P= 0.4647 in back skins of 3 mice) and 22 days (two-tailed, paired T-test, for lymphatic network length P= 0.7242 in ears of 4 mice and P= 0.7215 in back skins of 4 mice, for average lymphatic vessel diameter P= 0.7499 in ears of 4 mice and P= 0.0027 in back skins of 4 mice, for number of branching points P= 0.0350 in ears of 4 mice and P= 0.0668 in back skins of 4 mice) after the injections. Data are represented as mean and SEM from intradermally injected ears (c) and back skin (d) of 3 (2 days) and 4 (22 days) mice in each group.

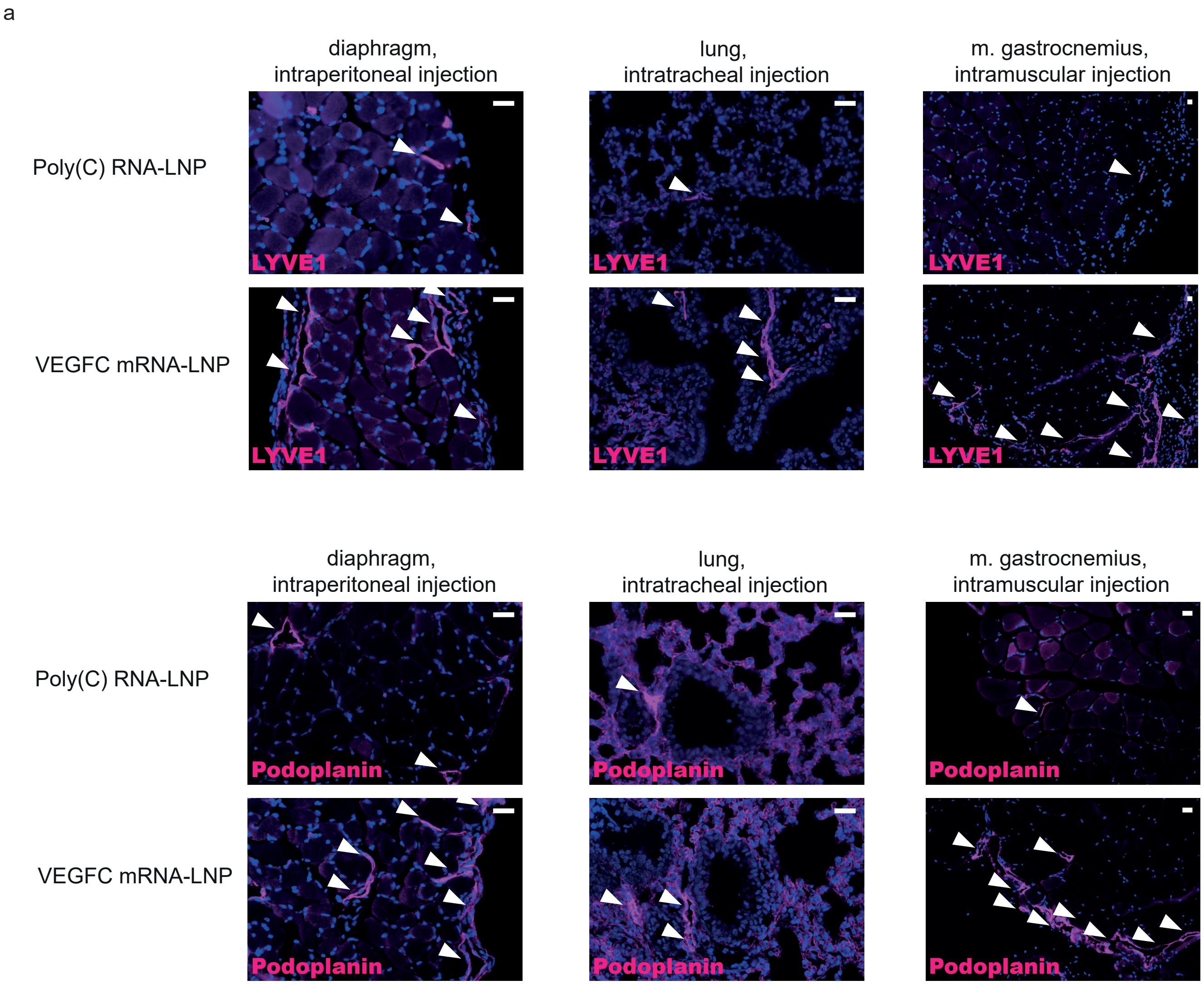

Supplementary Figure 5. VEGFC mRNA-LNP induces lymphangiogenesis in various organs.
(a) Monitoring the effect of 1 µg of Poly(C) and VEGFC mRNA-LNPs on lymphatic growth in C57Bl/6 mice in the diaphragm 22 days after intraperitoneal injection, in the lungs 22 days after intratracheal treatment and in the musculus gastrocnemius 22 days after intramuscular injection. Representative images with anti-LYVE1 and anti-Podoplanin immunostaining of 3-16 mice are shown by paraffin-based histology (bars, 50 µm). Arrows indicate LYVE1 or Podoplanin positive lymphatic vessels. All cell nuclei are labelled with DAPI (blue) in paraffin-embedded tissues.

Supplementary Figure 6
Szőke et al.

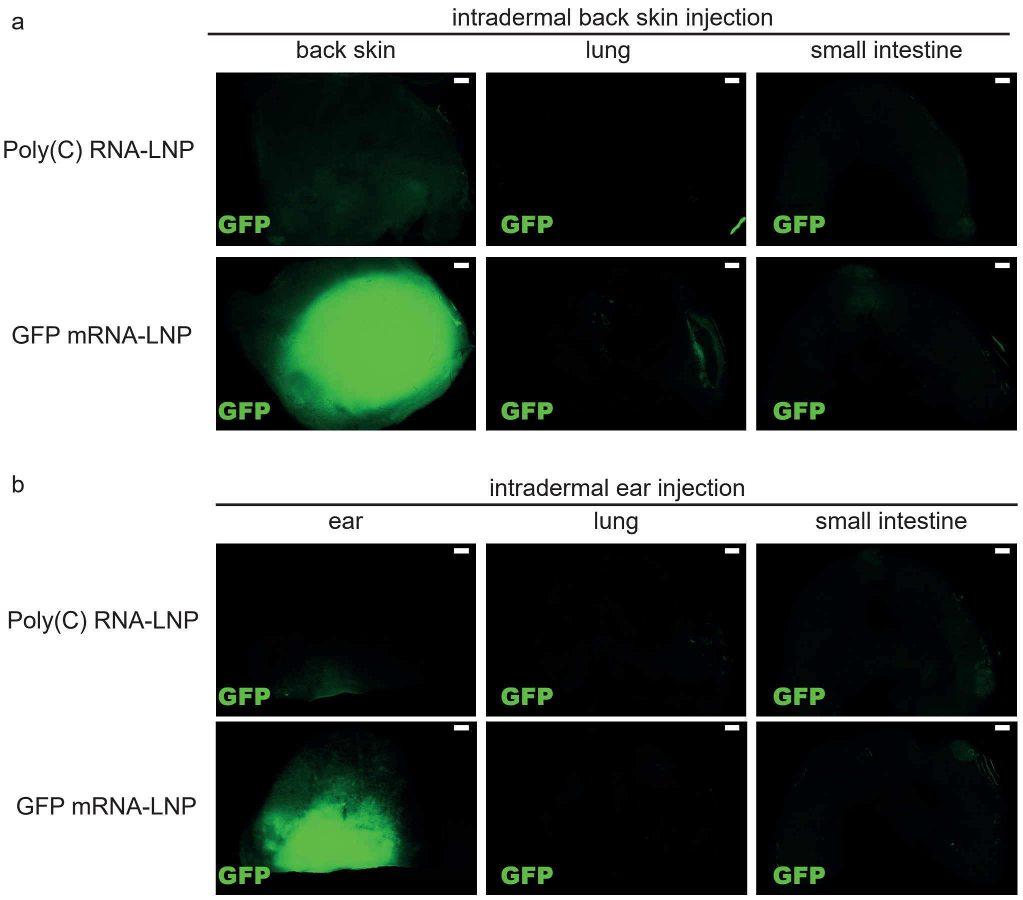

Supplementary Figure 6. Intradermal administration of GFP mRNA-LNPs results in protein production at the injection site.
(a) The back skin of C57Bl/6 mice were intradermally injected with 1 μg of Poly(C) or GFP mRNA-LNPs and GFP protein expression in the back skin, lungs and small intestines was monitored 4 hours post injection (bars, 1000 μm). Representative images of 3 mice per group are shown. **(b)** Ears of C57Bl/6 mice were intradermally injected with 0.2 μg of Poly(C) or GFP mRNA-LNPs and GFP protein expression in the ear, lung and small intestine was monitored 4 hours post injection. Representative images of 3 mice are shown by whole-mount fluorescent stereo microscopy (bars, 1000 μm).

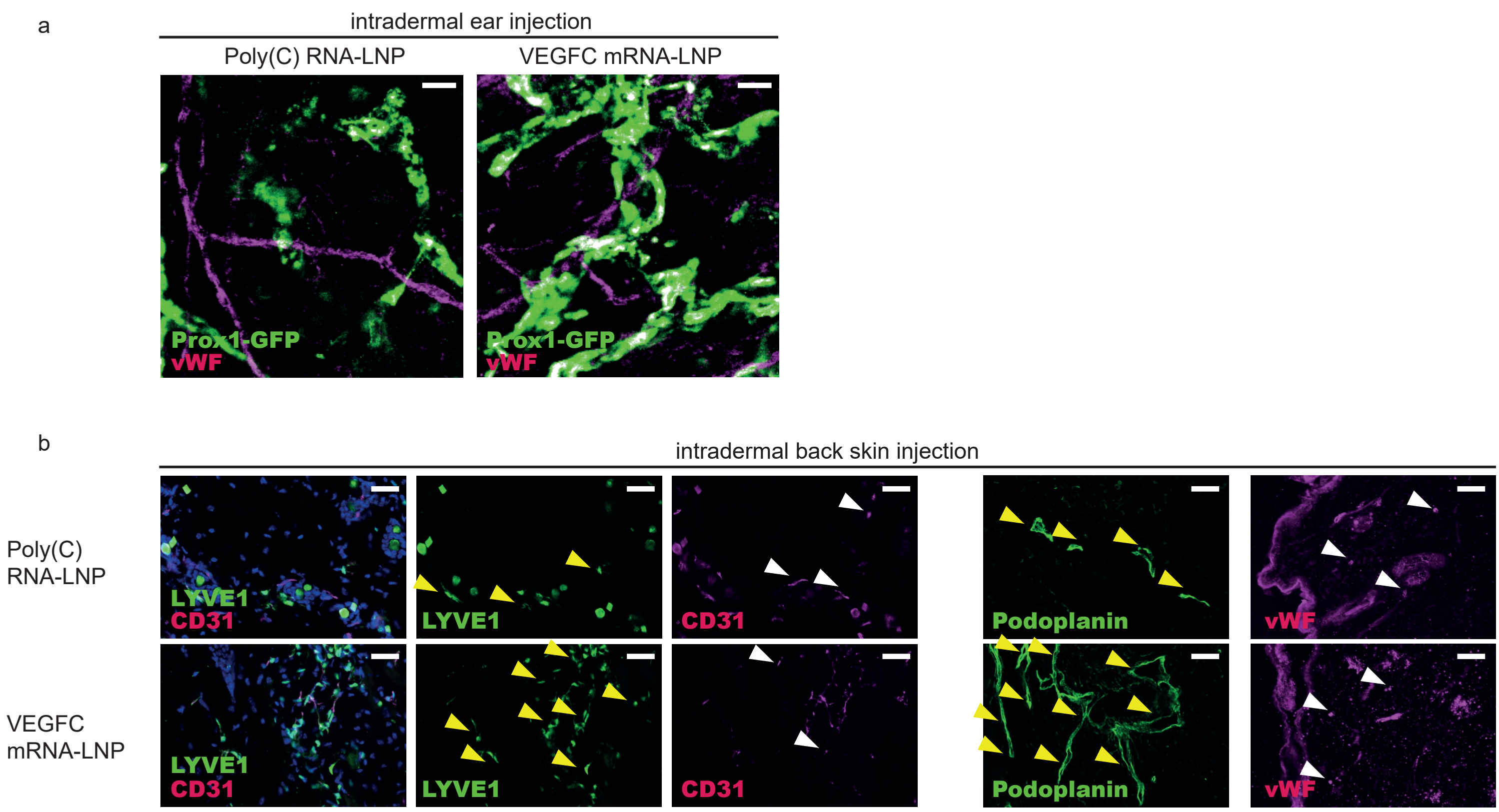

Supplementary Figure 7. No significant blood vessel proliferation was observed after intradermal administration of VEGFC mRNA-LNPs in the ear and back skin compared to Poly(C) RNA-LNP.
(a) 0.2 µg of Poly(C) or VEGFC mRNA-LNPs were intradermally injected into the ear of *Prox1*^{GFP} mice and the growth of blood and lymphatic vessels was examined 22 days after the injection. Representative confocal images of Prox1-GFP, anti-vWF stained samples are shown of 6 mice per group (bars, 50 µm). **(b)** 5 µg of Poly(C) or VEGFC mRNA-LNPs were intradermally injected into the back skin of *Prox1*^{GFP} mice and the growth of blood and lymphatic vessels was analyzed 22 days after the injection. Representative images of anti-CD31, anti-LYVE1, anti-Podoplanin and anti-vWF stained paraffin embedded samples are shown of 5 mice per group (bars, 50 µm). Yellow arrows indicate LYVE1 positive lymphatic vessels, white arrows indicate CD31 positive and LYVE1 negative blood vessels. All cell nuclei are labelled with DAPI (blue) in paraffin-embedded tissues.

a

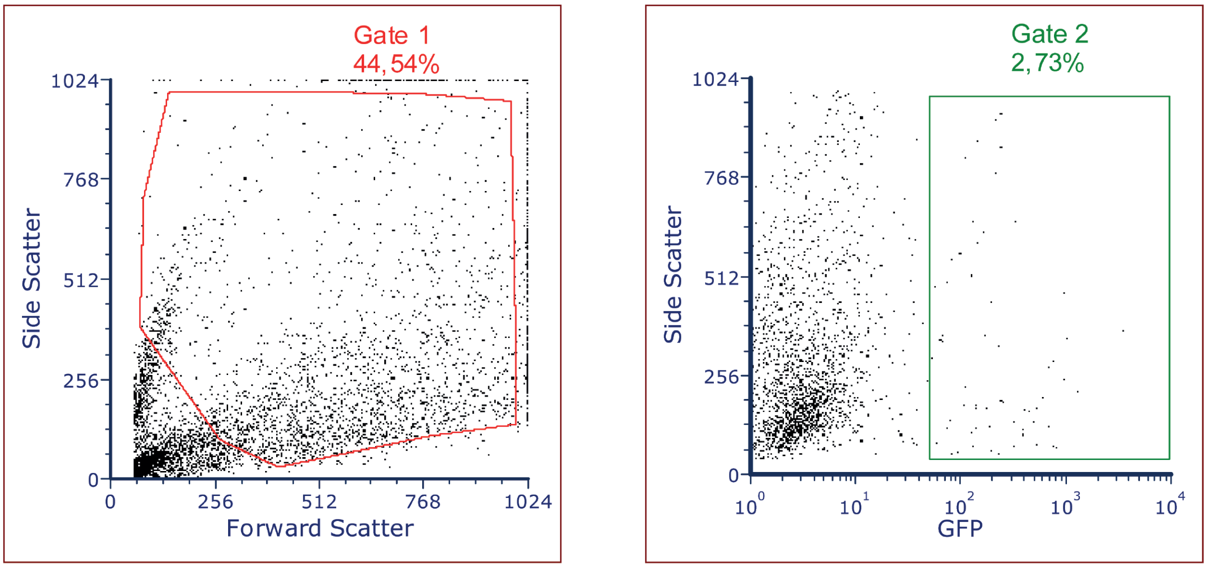

b

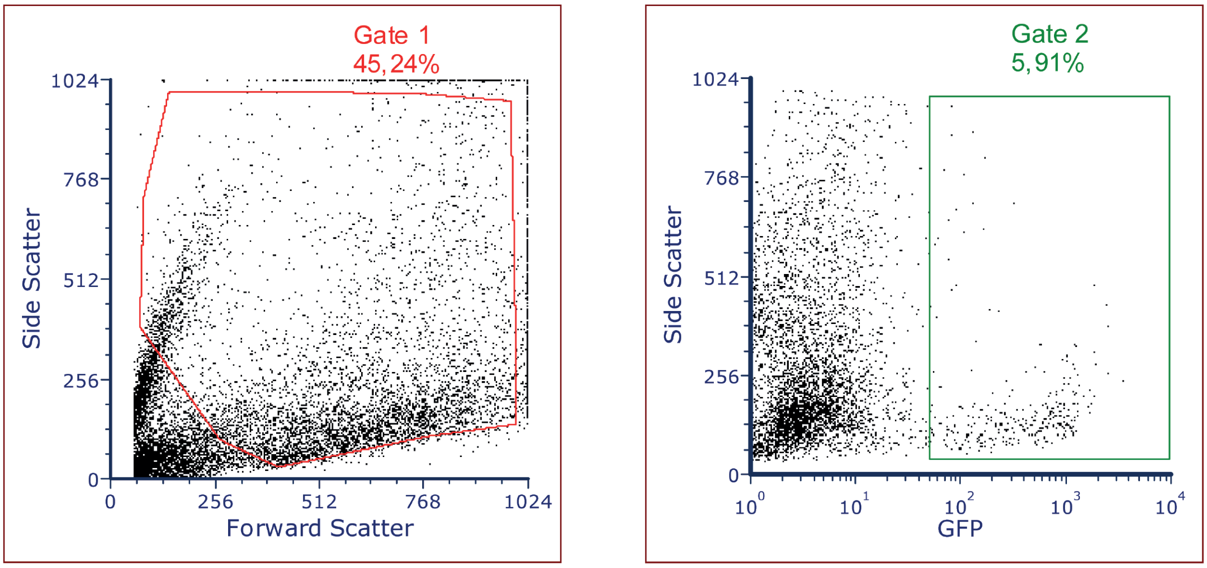

Supplementary Figure 8. Examples of gating strategy

(a-b) Gating strategy for flow cytometry of 1 µg Poly(C) RNA-LNP (a) and 1 µg VEGFC mRNA-LNP (b) injected ear of *Prox1*^{GFP} mice.

**Name of primer**	**Sequence of primer**
Prox1^{GFP} forward	5’ - GAT GTG CCA TAA ATC CCA GAG CCT AT – 3’
Prox1^{GFP} reverse	5’ – GGT CGG GGT AGC GGC TGA A – 3’
Flt4-CreER^{T2} knock in-specific forward	5’ – GGCTGGACCAATGTAAATATTG – 3’
Flt4-CreER^{T2} knock in-specific reverse	5’ – CATCATCGAAGCTTCACTG – 3’
Flt4 wild type-specific forward	5’ – CACTATGCTCCGTGTCTTG – 3’
Flt4 wild type-specific reverse	5’ – GTGACTCTCAGACATATG – 3’
iDTR$^{fl/fl}$ common forward	5’ – AAA GTC GCT CTG AGT TGT TAT – 3’
iDTR$^{fl/fl}$ wild type-specific reverse	5’ – GGA GCG GGA GAA ATG GAT ATG – 3’
iDTR$^{fl/fl}$ allele-specific reverse	5’ – CAT CAA GGA AAC CCT GGA CTA CTG – 3’

Supplementary Table 1. Primer sets used for genotyping.
