# Supplementary material for: Nucleoside-modified VEGFC mRNA induces organ-specific lymphatic growth and reverses experimental lymphedema
Source: Nat Commun. 2021 Jun 8;12:3460. doi: 10.1038/s41467-021-23546-6 (PMC8187400; doi:10.1038/s41467-021-23546-6)
Supplement: Supplementary file 2 — Reporting Summary [file 41467_2021_23546_MOESM2_ESM.pdf]

nature research

Corresponding author(s): Zoltán Jakus, MD, PhD
Norbert Pardi, PhD

Last updated by author(s): Feb 17, 2021

Reporting Summary

Nature Research wishes to improve the reproducibility of the work that we publish. This form provides structure for consistency and transparency in reporting. For further information on Nature Research policies, seeAuthors & Referees and theEditorial Policy Checklist .

Statistics

For all statistical analyses, confirm that the following items are present in the figure legend, table legend, main text, or Methods section.

n/a	Confirmed	
☐	☒	The exact sample size (*n*) for each experimental group/condition, given as a discrete number and unit of measurement
☐	☒	A statement on whether measurements were taken from distinct samples or whether the same sample was measured repeatedly
☐	☒	The statistical test(s) used AND whether they are one- or two-sided<br>*Only common tests should be described solely by name; describe more complex techniques in the Methods section.*
☐	☒	A description of all covariates tested
☐	☒	A description of any assumptions or corrections, such as tests of normality and adjustment for multiple comparisons
☐	☒	A full description of the statistical parameters including central tendency (e.g. means) or other basic estimates (e.g. regression coefficient) AND variation (e.g. standard deviation) or associated estimates of uncertainty (e.g. confidence intervals)
☐	☒	For null hypothesis testing, the test statistic (e.g. *F*, *t*, *r*) with confidence intervals, effect sizes, degrees of freedom and *P* value noted<br>*Give P values as exact values whenever suitable.*
☒	☐	For Bayesian analysis, information on the choice of priors and Markov chain Monte Carlo settings
☒	☐	For hierarchical and complex designs, identification of the appropriate level for tests and full reporting of outcomes
☒	☐	Estimates of effect sizes (e.g. Cohen's *d*, Pearson's *r*), indicating how they were calculated

Our web collection on statistics for biologists contains articles on many of the points above.

Software and code

Policy information about availability of computer code

Data collection

The following software programs were used to collect the data in this study:
NIS-Elements Imaging Software (Nikon) (version: BR 4.60.00)
BD CellQuest Pro (Becton Dickinson) (version: 6.1)
LAS X Software (Leica) 7.4.2.18368

Data analysis

The following software programs were used to analyze the data in this study:
NIS-Elements Imaging Software (Nikon) (version: BR 4.60.00)
NIH FIJI image processing software (versions 1.52i -1.53f)
Adobe Photoshop CS6 (Adobe) (version 16.0.0)
FCS Express 6 Flow Cytometry Software (De Novo Software) (version: 6.06.0033)
Graphpad Prism 7.03 (GraphPad Software Inc)
Excel 2018 (Microsoft)

For manuscripts utilizing custom algorithms or software that are central to the research but not yet described in published literature, software must be made available to editors/reviewers. We strongly encourage code deposition in a community repository (e.g. GitHub). See the Nature Research guidelines for submitting code & software for further information.

Data

Policy information about availability of data

All manuscripts must include a data availability statement. This statement should provide the following information, where applicable:

- Accession codes, unique identifiers, or web links for publicly available datasets
- A list of figures that have associated raw data
- A description of any restrictions on data availability

All the data supporting the findings of this study are available within the article and its supplementary information files and from the corresponding authors upon

reasonable request. A reporting summary for this article is available as a Supplementary Information file.

Field-specific reporting

Please select the one below that is the best fit for your research. If you are not sure, read the appropriate sections before making your selection.

- [x] Life sciences
- [ ] Behavioural & social sciences
- [ ] Ecological, evolutionary & environmental sciences

For a reference copy of the document with all sections, see nature.com/documents/nr-reporting-summary-flat.pdf

Life sciences study design

All studies must disclose on these points even when the disclosure is negative.

Sample size	No statistical analysis was performed to predetermine sample sizes. Sample sizes were chosen in accordance with the standard protocols of the field. Groups were arranged to include at least 3 individual samples to analyze the phenotypes and perform statistical analysis. We used statistical analysis consistent with the sample size for each experiment and we found sufficient statistical power between groups with the noted number of samples.
Data exclusions	No samples were excluded from the analysis.
Replication	Experiments were performed by using several mice of each condition. All experiments were repeated several times as it is indicated in the figure legends.
Randomization	Mice were used from different cages in the same experimental group to assure randomization.
Blinding	All investigators performing flow cytometry, Western Blot or ELISA assays were blinded for the sample origin.<br><br>Investigators performing microscopic imaging and quantification of microscopic images were blinded for treatment and sample origin.<br><br>In the genetic lymphedema model the thickness of the paw was assessed by spring loaded custom caliper (Kroeplin), and visible clinical signs of edema formation were scored on a 0 –10 scale by two investigators blinded for the treatment of the mice.<br><br>Investigators performing experiments monitoring lymphatic function in vivo were blinded for the treatment of the mice.

Reporting for specific materials, systems and methods

We require information from authors about some types of materials, experimental systems and methods used in many studies. Here, indicate whether each material, system or method listed is relevant to your study. If you are not sure if a list item applies to your research, read the appropriate section before selecting a response.

Materials & experimental systems

n/a	Involved in the study	
[ ]	[x]	Antibodies
[ ]	[x]	Eukaryotic cell lines
[x]	[ ]	Palaeontology
[ ]	[x]	Animals and other organisms
[x]	[ ]	Human research participants
[x]	[ ]	Clinical data

Methods

n/a	Involved in the study	
[x]	[ ]	ChIP-seq
[ ]	[x]	Flow cytometry
[x]	[ ]	MRI-based neuroimaging

Antibodies

Antibodies used	The following primary and secondary antibodies were used for immunostaining: anti-LYVE1 (R&D systems, AF2125), anti-CD31 (R&D systems, mab3628), anti-von Willebrand Factor (anti-vWF) (Sigma-Aldrich, F3520), anti-Podoplanin (R&D systems, AF3244 and Abcam, ab92319), anti-α-SMA (Abcam, ab124964), anti-Collagen I antibody (Abcam, ab34710) and anti-Ly6G/Ly6C (anti-GR1) (BD Pharmingen, 550291, clone: RB6-8C5). Anti-goat, anti-rabbit, anti-hamster and anti-rat secondary antibodies were used conjugated to Alexa Fluor 488, 568 and 594 (Life Technologies, anti-goat AF488: A11055, anti-rat AF568: A11077, anti-mouse AF488: A11029, anti-mouse AF568: A11031, anti-mouse AF594: A32744, anti-rabbit AF488: AF11034, anti-rabbit AF568 AF11036).<br><br>The following primary and secondary antibodies were used for immunoblotting: anti-GFP (Abcam, ab6673); anti-VEGFC (R&D Systems, AF752); anti-β-actin (Sigma-Aldrich, A2228, Clone: AC-74); anti-mouse-HRP (GE Healthcare, NA931); anti-goat-HRP (Life Technologies, A16005).

The expressed amount of VEGFC was also determined with VEGFC Rat ELISA Kit (Invitrogen, BMS626-2).

The following primary and secondary antibodies were used for whole mount immunostaining: anti-von Willebrand Factor (anti-vWF) (Sigma-Aldrich, F3520); anti-GFP (Abcam, ab6673); anti-rat AF568 (Life Technologies, A11077); anti-goat AF488 (Life Technologies, A11055).

The following antibodies were used for flow cytometry: PE anti-mouse LYVE-1 (R&D Systems, FAB2125P), Alexa Fluor 488 anti-mouse CD206 (BioLegend, BZ-141710), Alexa Fluor 647 Rat Anti-Mouse CD3 (BD Biosciences, 557869), PE Rat Anti-Mouse CD45R/B220 (BD Biosciences, 553090), PerCP-Cy5.5 anti-mouse CD11b (BD Biosciences, 550990) PE anti-mouse CD45 (Biolegend, 103105, Clone: 30-F11), FITC anti-mouse Ly6G/C Antibody (BD Biosciences, 553127, Clone: RB6-8C5).

The following dilutions were used during our study. For Western blot, we used 1:1 000 dilution for primary and 1:10 000 dilution for secondary antibodies. For immunhistochemistry and whole mount immunostaining we used 1:100 dilution for primary and 1:250 for secondary antibodies. For FACS analysis we used 1:200 dilution for all antibodies.

Validation

All primary antibodies were validated using appropriate controls.

The following antibody anti-LYVE1 (R&D systems, AF2125) has been used by at least 43 publications. The manufacturer also provides antibody testing data. https://www.rndsystems.com/products/mouse-lyve-1-antibody_af2125

The following antibody anti-CD31 (R&D systems, mab3628) has been used by at least 2 publications. The manufacturer also provides antibody testing data. https://www.rndsystems.com/products/mouse-cd31-pecam-1-antibody-693102_mab3628

The following antibody anti-von Willebrand Factor (anti-vWF) (Sigma-Aldrich, F3520) has been used by at least 78 publications. The manufacturer also provides antibody testing data. https://www.sigmaaldrich.com/catalog/product/sigma/f3520

The following antibody anti-Podoplanin (R&D systems, AF3244) has been used by at least 32 publications. The manufacturer also provides antibody testing data. https://www.rndsystems.com/products/mouse-podoplanin-antibody_af3244

The following antibody anti-Podoplanin (Abcam, ab92319) has been used by at least 4 publications. The manufacturer also provides antibody testing data. https://www.abcam.com/podoplanin--gp36-antibody-811-ab92319.html

The following antibody anti-α-SMA (Abcam, ab124964) has been used by at least 118 publications. The manufacturer also provides antibody testing data. https://www.abcam.com/alpha-smooth-muscle-actin-antibody-epr5368-ab124964.html

The following antibody anti-Collagen I antibody (Abcam, ab34710) has been used by at least 835 publications. The manufacturer also provides antibody testing data. https://www.abcam.com/collagen-i-antibody-ab34710.html

The following antibody anti-Ly6G/Ly6C (anti-GR1) (BD Pharmingen, 550291, clone: RB6-8C5) has been used by at least 11 publications. The manufacturer also provides antibody testing data. https://www.bdbiosciences.com/us/reagents/research/antibodies-buffers/immunology-reagents/anti-mouse-antibodies/cell-surface-antigens/purified-rat-anti-mouse-ly-6g-and-ly-6c-rb6-8c5/p/550291

The following antibody anti-GFP (Abcam, ab6673) has been used by at least 310 publications. The manufacturer also provides antibody testing data. https://www.abcam.com/gfp-antibody-ab6673.html

The following antibody anti-VEGFC (R&D Systems, AF752) has been used by at least 12 publications. The manufacturer also provides antibody testing data. https://www.rndsystems.com/products/human-vegf-c-antibody_af752

For the following antibody anti-β-actin (Sigma-Aldrich, A2228, Clone: AC-74) the manufacturer provides antibody testing data. https://www.sigmaaldrich.com/catalog/product/sigma/a2228

For the following antibody anti-mouse-HRP (GE Healthcare, NA931) the manufacturer provides antibody testing data. https://www.sigmaaldrich.com/catalog/product/sigma/gena9311ml

The following antibody anti-goat-HRP (Life Technologies, A16005) has been used by at least 4 publications. The manufacturer also provides antibody testing data. https://www.thermofisher.com/antibody/product/Donkey-anti-Goat-IgG-H-L-Cross-Adsorbed-Secondary-Antibody-Polyclonal/A16005

The following antibody anti-rat AF568 (Life Technologies, A11077) has been used by at least 85 publications. The manufacturer also provides antibody testing data. https://www.thermofisher.com/antibody/product/Goat-anti-Rat-IgG-H-L-Cross-Adsorbed-Secondary-Antibody-Polyclonal/A-11077

The following antibody anti-goat AF488 (Life Technologies, A11055) has been used by at least 230 publications. The manufacturer also provides antibody testing data. https://www.thermofisher.com/order/genome-database/details/antibody/A-11055.html

The following antibody PE anti-mouse LYVE-1 (R&D Systems, FAB2125P) has been used by at least 3 publications. The

manufacturer also provides antibody testing data. https://www.rndsystems.com/products/mouse-lyve-1-pe-conjugated-antibody-223322_fab2125p

The following antibody Alexa Fluor 488 anti-mouse CD206 (BioLegend, BZ-141710) has been used by at least 14 publications. The manufacturer also provides antibody testing data. https://www.biolegend.com/en-us/products/alexa-fluor-488-anti-mouse-cd206-mmr-antibody-7426

The following antibody Alexa Fluor 647 Rat Anti-Mouse CD3 (BD Biosciences, 557869) has been used by at least 2 publications. The manufacturer also provides antibody testing data. https://www.bdbiosciences.com/in/applications/research/t-cell-immunology/th-1-cells/surface-markers/mouse/alexa-fluor-647-rat-anti-mouse-cd3-molecular-complex-17a2/p/557869

The following antibody PE Rat Anti-Mouse CD45R/B220 (BD Biosciences, 553090) has been used by at least 17 publications. The manufacturer also provides antibody testing data. https://www.bdbiosciences.com/us/applications/research/stem-cell-research/hematopoietic-stem-cell-markers/mouse/negative-markers/pe-rat-anti-mouse-cd45rb220-ra3-6b2/p/553090

The following antibody PerCP-Cy5.5 anti-mouse CD11b (BD Biosciences, 550990) has been used by at least 10 publications. The manufacturer also provides antibody testing data. https://www.bdbiosciences.com/us/applications/research/stem-cell-research/mesenchymal-stem-cell-markers-bone-marrow/mouse/negative-markers/percp-cy55-rat-anti-cd11b-m170/p/550993

The following antibody PE anti-mouse CD45 (Biolegend, 103105, Clone: 30-F11) has been used by at least 63 publications. The manufacturer also provides antibody testing data. https://www.biolegend.com/en-us/products/pe-anti-mouse-cd45-antibody-100

The following antibody FITC anti-mouse Ly6G/C Antibody (BD Biosciences, 553127, Clone: RB6-8C5) has been used by at least 8 publications. The manufacturer also provides antibody testing data. https://www.bdbiosciences.com/us/reagents/research/antibodies-buffers/immunology-reagents/anti-mouse-antibodies/cell-surface-antigens/fitc-rat-anti-mouse-ly-6g-and-ly-6c-rb6-8c5/p/553127

Eukaryotic cell lines

Policy information about cell lines

Cell line source(s)	HEK 293T cells from ATCC (Suppl. Figs. 1, 2).
Authentication	The cell lines were not authenticated.
Mycoplasma contamination	The cell lines were negative in mycoplasma testing.
Commonly misidentified lines (See ICLAC register)	None

Animals and other organisms

Policy information about studies involving animals; ARRIVE guidelines recommended for reporting animal research

Laboratory animals	6-12 week-old C57Bl/6 wild type mice were purchased from commercial sources (Envigo and the Hungarian National Institute of Oncology). 6-12 week-old Prox1GFP BAC lymphatic reporter animals obtained from the Mutant Mouse Regional Resource Centers (MMRRC) were maintained in heterozygous form. To eliminate the lymphatic endothelial cells in a genetic experimental lymphedema model the Flt4-CreERT2; iDTRflox/flox strain on the C57Bl/6 background was used (6-24 week-old animals). Experimental animals were housed in either specific pathogen free or conventional animal facilities. Both male and female mice were used. Experimental animals were housed in either specific pathogen free or conventional animal facilities between 18-22°C, 45% humidity and 12/12 hours dark-light cycles.
Wild animals	No wild animals were used in this study.
Field-collected samples	No field-collected samples were used in this study.
Ethics oversight	All animal experiments were approved by the Animal Experimentation Review Board of the Semmelweis University and the Government Office for Pest County (Hungary) under license number PE/EA/148-4/2018 and PEI/001/404-8/2015 with approval for the use of genetically modified organisms under license number TMF/305-63/2015 issued by the Ministry of Agriculture (Hungary).

Note that full information on the approval of the study protocol must also be provided in the manuscript.

Flow Cytometry

Plots

Confirm that:

☐ The axis labels state the marker and fluorochrome used (e.g. CD4-FITC).

☒ The axis scales are clearly visible. Include numbers along axes only for bottom left plot of group (a 'group' is an analysis of identical markers).

☐ All plots are contour plots with outliers or pseudocolor plots.

☒ A numerical value for number of cells or percentage (with statistics) is provided.

Methodology

Sample preparation	Ear samples from C57Bl/6 and Prox1GFP mice treated with Poly(C) and VEGFC mRNA-LNPs were collected and cut into small pieces, then digested with the Liberase II kit (Roche) with Eppendorf Thermomixer at 1,400 rpm for 1 h at 37°C. Single-cell suspensions were obtained by passing the digest through a 70-µm cell strainer (BD). LECs were identified as LYVE1-positive, leukocytes as CD45-positive, granulocytes as Ly6G/C-positives, T lymphocytes as CD3-positive, B lymphocytes as B220-positive and monocytes as Ly6G/C negative and CD11b positive cells.
Instrument	BD Biosciences FACSCalibur cytometer
Software	Data were collected using BD CellQuest Pro (Becton Dickinson) (version: 6.1) software. Analysis was performed using FCS Express 6 Flow Cytometry Software (De Novo Software) (version: 6.06.0033).
Cell population abundance	We did not sort any cell lines of the samples. Each sample was treated with the same method and the appropriate controls were used in each experiments.
Gating strategy	For all experiment, the gating strategy involved gating on cell popolation followed by the staining of interest. Examples of gating strategy is provided in the Supplementary Information (Supplementary Figure 8).

☒ Tick this box to confirm that a figure exemplifying the gating strategy is provided in the Supplementary Information.
